# Long-term outcome of autologous haematopoietic stem cell transplantation in patients with systemic sclerosis: a comparison with patients treated with rituximab and with traditional immunosuppressive agents

**DOI:** 10.1186/s13075-024-03408-4

**Published:** 2024-10-23

**Authors:** Nicoletta Del Papa, Silvia Cavalli, Andrea Rindone, Francesco Onida, Giorgia Saporiti, Antonina Minniti, Maria Rosa Pellico, Claudia Iannone, Giorgia Trignani, Nicoletta D’Angelo, Manuel Sette, Raffaella Greco, Claudio Vitali, Roberto Caporali

**Affiliations:** 1 Scleroderma Clinic, UOC Clinica Reumatologica, ASST Pini-CTO, Milano, Italy; 2 Department of Clinical Sciences and Community Health, Università degli Studi di Milano, Milano, Italy; 3 Department of Oncology and Onco-Hematology, Università degli Studi di Milano, Milano, Italy; 4 Ospedale Fatebenefratelli e Oftalmico, Oncoematologia, Milano, Italy; 5 Hematology Unit, Fondazione IRCCS Ca’ Granda Ospedale Maggiore Policlinico, Milano, Italy; 6 Unit of Hematology and Bone Marrow Transplantation, IRCCS San Raffaele Hospital, Vita- Salute San Raffaele University, Milano, Italy; 7 Rheumatology Outpatient Clinic, Mater Domini Humanitas Hospital, Castellanza, Italy

**Keywords:** Autologous haematopoietic stem cell transplantation, Systemic sclerosis, Rituximab, Immunosuppressive therapy

## Abstract

**Background:**

Autologous haematopoietic stem cell transplantation (AHSCT) is more effective than conventional immunosuppressive therapies (CIT) in improving the outcome of patients with rapidly progressive diffuse cutaneous systemic sclerosis (dcSSc). So far, there is still a paucity of data comparing AHSCT with rituximab (RTX). Aim of the study is to retrospectively compare, in patients with dcSSc, the effectiveness of AHSCT with that of RTX and CIT.

**Methods:**

Thirty-five dcSSc AHSCT-treated patients were compared with 29 and 36 matched cases treated with RTX and CIT, respectively. The patients were followed up for 5 years by assessing selected outcome measures every year. Overall survival, modified Rodnan skin score (mRSS), lung function tests (FVC and DLCO), and the revised EUSTAR Activity Index (REAI) were the outcome measures chosen to evaluate the therapy efficacy.

**Results:**

AHSCT was significantly more effective than RTX and CIT in prolonging survival, inducing a rapid reduction of the mRSS and REAI and maintaining the baseline level of lung function tests for a longer time. RTX therapy was also superior to CIT in reducing REAI, mRSS and in saving lung function.

**Conclusion:**

AHSCT is more effective than both RTX and CIT in prolonging survival and inducing prolonged remission in patients with rapidly progressive dcSSc.

## Introduction

Systemic sclerosis (SSc) is a rare systemic autoimmune disease characterized by the accumulation of extracellular collagen matrix in target organs and tissues, such as skin, lung, gut and heart [1]. The clinical spectrum of SSc is largely heterogeneous, but usually two distinct forms are recognized., i.e., the limited cutaneous (lc) and the diffuse cutaneous (dc) SSc. The two variants strongly differ in the extension of cutaneous involvement, type and severity of internal organ involvement, and prognosis. The survival of patients with SSc has improved during the last decades, with an overall 10-year survival ranging from 63 to 75.5% [2, 3], interstitial lung disease, pulmonary artery hypertension and cardiac issues being the leading causes of death [4]. However, rapidly progressive form of the disease can lead to a 5-year mortality rate around 35% [5]. This has pushed the clinicians to treat this kind of patients in a more aggressive manner. For many years the standard of care in this subset of patients has been limited to the use of immunosuppressive therapy such as methotrexate (MTX), cyclophosphamide (CYC), azathioprine (AZA) or mycofenolate mofetil (MMF). Contradictory results have been obtained with these agents due to the different modality of their use in series of patients which were not comparable [6].

The arrival of newer target therapies has opened new possibilities for the treatment of SSc as a whole and for specific different pathological features. Randomized controlled trials (RCT) and open labeled studies have shown that tocilizumab and rituximab (RTX) may be effective in modulating the inflammatory process underlying the disorder, and nintedanib in lowering the fibrosis progression in some target organs such as the lung [7–10].

In rapidly progressive cases of dcSSc, autologous haematopoietic stem cell transplantation (AHSCT) has been recently considered as a standard-of-care therapeutic option [11, 12]. This procedure was first assessed in open label trials and its effectiveness, in lowering the disease progression and improving the survival rate, was then confirmed by three RCTs [13, 14].

To our knowledge, to date, no studies have been published comparing the long-term outcome in patients with only rapidly progressive dcSSc treated with AHSCT with those who received RTX and conventional immunosuppressive therapies (CIT).

In the present retrospective study our aim was to compare different therapeutical regimens (AHSCT vs. patients treated RTX vs. historical group of patients who received CIT), in patients with rapidly progressive form of dcSSc.

## Patients and methods

### Patients

All the patients included in this retrospective study and treated with the three different therapeutic regimens had a rapidly progressive dcSSc, characterized by a modified Rodnan skin score (mRSS) ≥ 14 at the baseline observation and a disease duration less than 4 years. They met the 1993 American College of Rheumatology and, when retrospectively evaluated, also the 2013 ACR-EULAR criteria [15, 16].

For the intention of the present study and to more precisely assess the disease response to the different treatment regimens within the three groups, we have selected specific outcome measures that can provide quantitative or semi-quantitative assessment of different disease features. Specifically, we considered (*i*) the severity of skin involvement measured by the mRSS. Reduction of at least 5 points or of 25% or more of the baseline values of this score were taken into account to define the improvement of skin involvement [17]; (*ii*) the degree of lung function impairment was assessed by forced vital capacity (FVC) and diffusion lung of carbon monoxide (DLCO), expressed as a percentage of the predicted value. Reduction of FVC ≥ 10% alone and decline of FVC ≥ 10% or DLCO ≥ 15% were considered the lung function impairment defining the progression of lung involvement [18]; (*iii*) the overall assessment of disease activity, using the Revised EUSTAR Activity Index (REAI) scoring system. Values ≥ 2.5 were considered as indicative of high level of disease activity, and consequently the decline of this score below this value as an important achievement [19]. Finally, the disease-related mortality rate in the differently treated groups was also recorded during the follow-up and at the end of the study period.

The assessment of all these parameters was done once a year in the whole population of patients. Clinical monitoring of the disease features was also made every 3–6 months, according to the disease course in any single case.

### Transplanted patients

Between 2003 and 2019, 35 patients with rapidly progressive dcSSc underwent AHSCT in our Scleroderma Unit. Criteria for inclusion were a clinical activity score equal or over 2.5, according to the REAI [19], and a disease duration ≤ 4 years. This cut-off value for disease duration was chosen in agreement with that used in other studies, namely the Autologous Stem Cell Transplantation International Scleroderma (ASTIS) trial [20]. Candidates for AHSCT were considered those patients whose response to CIT was nil or very unsatisfactory. Previous unsuccessful immunosuppressive treatment included MTX (15–25 mg/week) in 17 patients, AZA in 4, CYC (1 g monthly for 2 months) in 4, MMF in 10, in any case associated with low-dose prednisone (≤ 7.5 mg/day).

Important co-morbidities and any pre-existing or current severe disease-related organ involvement, such as pulmonary arterial hypertension (detected by echocardiography and confirmed by right heart catheterization), scleroderma renal crisis, interstitial lung disease (ILD) with a DLCO under 70% of the predicted value, and scleroderma cardiopathy with an ejection fraction below 45%, were all considered exclusion criteria for AHSCT [21]. To have a correct selection, a complete clinical and instrumental work up was made before starting the transplantation procedure. The AHSCT procedure was performed similarly to what had been done in previous studies [20]. Previous unsuccessful immunosuppressive therapies were discontinued at least 1 month before the mobilization procedure. Thirty-four patients received conditioning with high-dose CYC (CYC 200 mg/kg and 7.5 mg/kg Thymoglobulin), 30 with and 2 without CD34 selection of the graft; two patients received a fludarabine-based cardiac-safe conditioning (rituximab 1000 mg, CYC 60 mg/kg and fludarabine 120 mg/m2).

None of the transplanted patients enrolled in this study was included in other studies and namely in the ASTIS trial, although some of the authors of the present study took part in that multicenter survey.

Additional clinical and demographic features of this group of patients are detailed in Table 1.

**Table 1:** Baseline demographic, clinical and serological features of the differently treated populations of patients

Characteristics	AHSCT (*N* = 35)	RTX (*N* = 29)	CIT (*N* = 36)
Age, median (range), years	44 (20–64)	47 (36–55)	44(19–62)
Female, n (%)	27 (77.14)	24 (82.76)	26 (72.22)
Duration of the disease, median (range), months	24 (10–48)	27 (15–54)	24 (6–48)
mRSS, median (range)	20 (15–32)	20 (16–24)	19 (14–32)
REAI, median (range)	6.00 (4.12–7.75)	6.75 (4.50–7.75)	6.00 (4.00–8.00)
FVC%, median (range)	89 (79–110)	89 (88–95)	87 (80–99)
EF%, median (range)	60 (57–65)	60 (55–68)	62 (45–76)
PAPs, median (range)	27 (25–29)	28 (25–33)	30 (28–32)
HRCT % of ILD extension, n° (%) of patients			
<5	14 (40.00)	12 (41.38)	14 (38.89)
5–20	21 (60.00)	16 (55.17)	20 (55.55)
>20	0 (0)	1 (3.44)	2 (5.55)
ANA positivity, n°, (%) of patients			
any pattern	35 (100)	29 (100)	36 (100)
homegeneous pattern	18 (51.43)	12 (41.38)	17 (47.22)
nucleolar pattern	12 (34.28)	9 (31.03)	13 (36,11)
speckled pattern	5 (14.28)	8 (27.58)	6 (16.66)
anti-Scl70 positivity, n (%)	32 (91.40)	26 (89.60)	26 (72.00)

Table 1. AHSCT: autologous haematopoietic stem cell transplantation, ANA: anti-nuclear antibody, CIT: conventional immunosuppressive therapies, EF: Ejection fraction, HRCT: high resolution computed tomography, ILD: interstitial lung disease, FVC: forced vital capacity, mRSS: modified Rodnan skin score, PAPs: systolic pulmonary artery pressure, REAI: revised European Activity Index, RTX: rituximab

### RTX-treated patients

From 2012 to 2019, 29 patients with rapidly progressive dcSSc were treated with RTX. This group of patients included those who had received RTX for at least 24 months and with no previous CYC treatment history.

The criteria for inclusion in this therapeutic group were having a rapidly progressive dcSSc with the same characteristics as the transplanted patients but to have refused or to be strongly puzzled when the AHSCT option was proposed. RTX treatment was given in courses once every 6 months so that each course would contain 2 doses (each of 1000 mg, intravenously administered) with two-week intervals, in combination with methyl-prednisolone (100 mg), antihistamine and paracetamol premedication. All patients had maintenance RTX infusions of 1 g every 6 months (median number 3, range 2–5). Seventeen patients (58%) also received another CIT concomitantly: MMF (8), MTX (9). Monthly IVIG was also administered initially in 3 patients (10%), while 12 patients (41%) were treated with RTX and steroids only.

More details on the demographic and clinical characteristics of these patients are reported in Table 1.

### Patients treated with immunosuppressive drugs

This is a historical group of 36 patients who were treated with CIT from 1991 to 2003 with the aim of stopping or reducing the clinical progression of their rapidly progressive dcSSc. These patients were treated with multiple immunosuppressive regimens, either sequentially or with combinations of different drugs. Overall, 25 of these patients received CYC (monthly infusions for 6 months, some of them also adjunctive infusions at 9 and 12 months), 18 received MTX (10–20 mg per week), 7 received MMF (2 or 3 gr per day), 18 received AZA (100–200 mg per day) and 3 received IVIG (400 mg/kg per day, 5 days per month for 6 months). None of these patients was treated with a unique therapeutical regimen during the entire follow up, but different CIT were used in double combination or in subsequent times. Considering this extreme inhomogeneity of the therapeutical regimens, it was decided to analyse the CIT-treated group as a whole.

Data on this group of CIT-treated and their comparison with data from 18 transplanted patients were the subject of a previous report from our group [22]. Additional details on this control population are reported in Table 1.

### Statistical analysis

Kaplan-Meier curves and log-rank test were used to compare overall survival observed in the different groups of patients during the 5-year follow-up. Hazard Ratio (HR) with 95% confidence interval (CI) were also computed. The same statistical approach was adopted to analyse the survival of baseline values of the mRSS. Reduction of this parameter of 5 or more points or of 25% or more in comparison with the baseline values was considered as indicative of improvement of skin involvement [17]. Progression of lung involvement was derived by the survival curve of FVC and FVC/DLCO in combination, where the above-mentioned overtime percentual changes of these lung function tests were considered [18]. Finally, the survival curve of REAI was derived and a decrease of this score under 2.5 was taken into account as an important decline of disease activity [19].

It is worth noting that FVC, mRSS and REAI are not independent variables since both FVC and the mRSS are included in the REAI scoring system. However, we decided to separately analyze these parameters since FVC and the mRSS may represent the mirror of some specific organ involvement (lung and skin, respectively), while REAI is a composite measure of global disease activity.

Overtime changes of mRSS and FVC in the three different groups were analyzed and compared to each other using generalized linear model with repeated measures. Sphericity of each variance was evaluated computing the ε value. Since this value in all the comparisons was < 0.75, a correction according to Greenhouse-Geisser method was made.

## Results

### Patients’ characteristics at the time of enrolment

Table 1 reports the main demographic and clinical characteristics of the enrolled patients subdivided into the three groups.

The 35 transplanted patients (27 females and 8 males) had a median age of 44 years (ranging from 20 to 64), a median disease duration of 24 months (ranging from 10 to 48), and a median baseline mRSS of 20.5 (ranging from 15 to 32). The median FVC was 89% of the predicted value, ranging from 79 to 110. At baseline, the high-resolution computed tomography (HRCT) was considered normal in 14 patients (41%), while it showed evidence of mild ILD in the remaining 21. As far as disease activity level in the AHSCT-treated group is concerned, all the enrolled patients had a median REAI score of 6.0 (ranging from 4.12 to 7.75).

Twenty-nine patients were treated with RTX. Their demographic and clinical features were almost completely identical to those of the AHSCT- and CIT-treated patients. The only slight difference was that the RTX-treated patients had a more restricted age range with respect to the patients in the other two groups, although the median age was the same (see Table 1).

The demographic and clinical findings of the group of 36 patients treated with CIT were also the same as those of the other two groups. The only difference is the lower prevalence of anti-topoisomerase-I antibodies (anti-Scl70) in this group. However, the difference is not statistically significant (see Table 1).

### Mortality and adverse effects after the AHSCT procedure, and during the CIT and RTX treatments

Following the AHSCT, one patient died from interstitial pneumonia at day 65, and another died immediately after transplantation procedure due to fulminant viral myocarditis, accounting for a transplant-related mortality of 5.7%. It is important to underline that these two AHSCT-related deaths happened in the early years in which our group experienced the transplantation procedure (2007 and 2008, respectively). None of the transplanted patients died later because of the more stringent enrolment criteria we adopted before deciding to apply the AHSCT procedure. The data from these patients were not considered when we analyzed the disease-related outcome variables in the AHSCT-treated patients. The adverse events observed during the whole transplantation procedure were not different, in terms of prevalence and severity, from those reported in previous similar studies [20, 22]. Namely, in the mobilization phase we observed six cases of fever of unknown origin, three case of mucositis and one case of haemorrhagic cystitis. During aplasia, we recorded ten cases of fever of unknown origin, eight cases of fever with positive blood culture and four cases of pneumonia. All the observed infections resolved thanks to adequate antibiotic treatment. In one case, we observed a transient reduction of left ventricular ejection fraction, and in another one a phase of arterial hypotension due to unexplained polyuria that required adequate re-hydration. No significant modifications of the SSc disease course were observed immediately after the mobilization phase and transplantation procedure.

No significant adverse event was observed in long-term follow-up after AHSCT and during the RTX and CIT treatment.

### Comparison of the survival curves of the considered outcome measures between AHSCT-treated patients and RTX and CIT treated patients

**Fig. 1:**
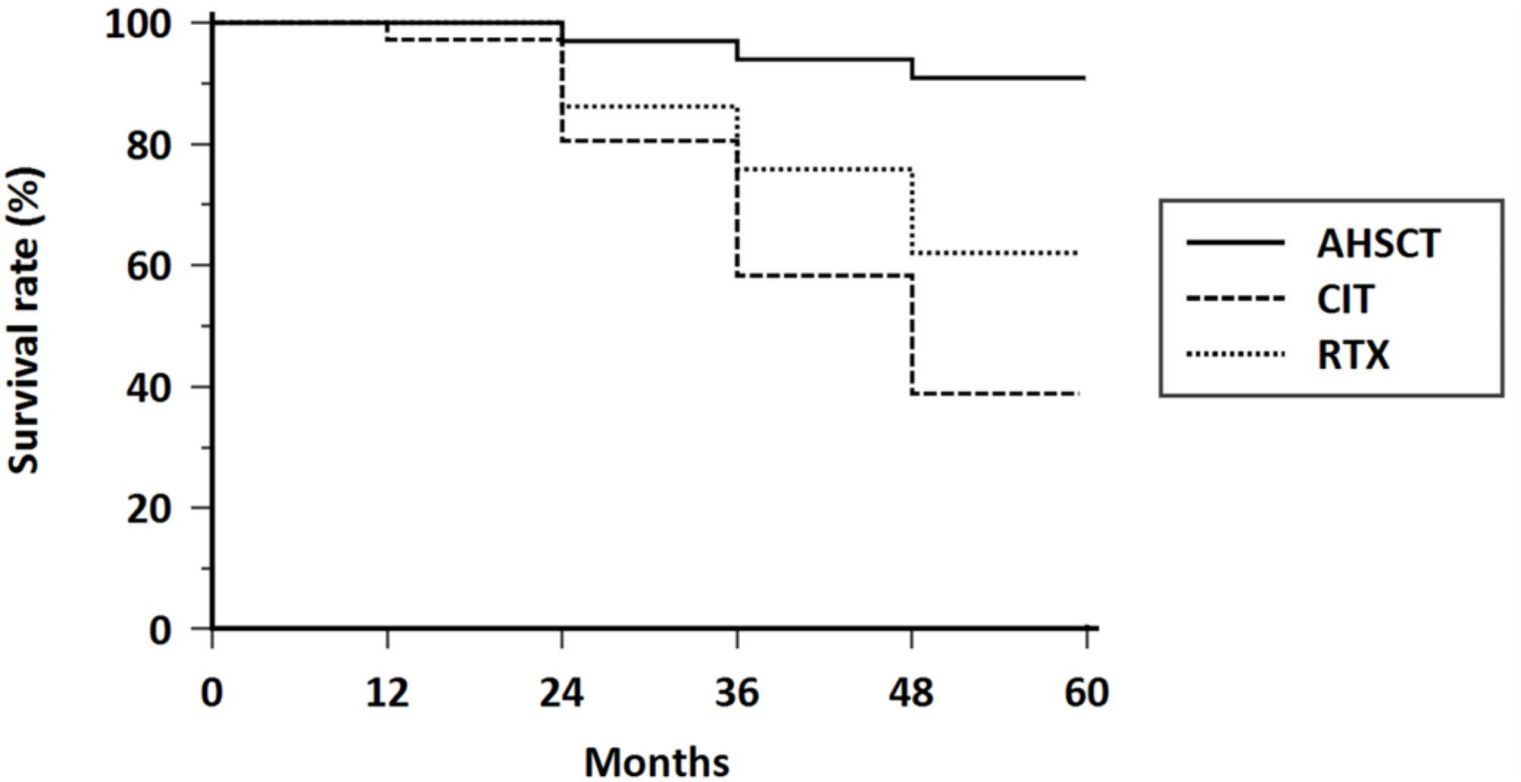
Overall survival rate analyzed by Kaplan-Meier curves in AHSCT-, RTX-, and CIT-treated patients. Detailed statistical results of log-rank test, HR and CI are reported in Table 2

**Fig. 2:**
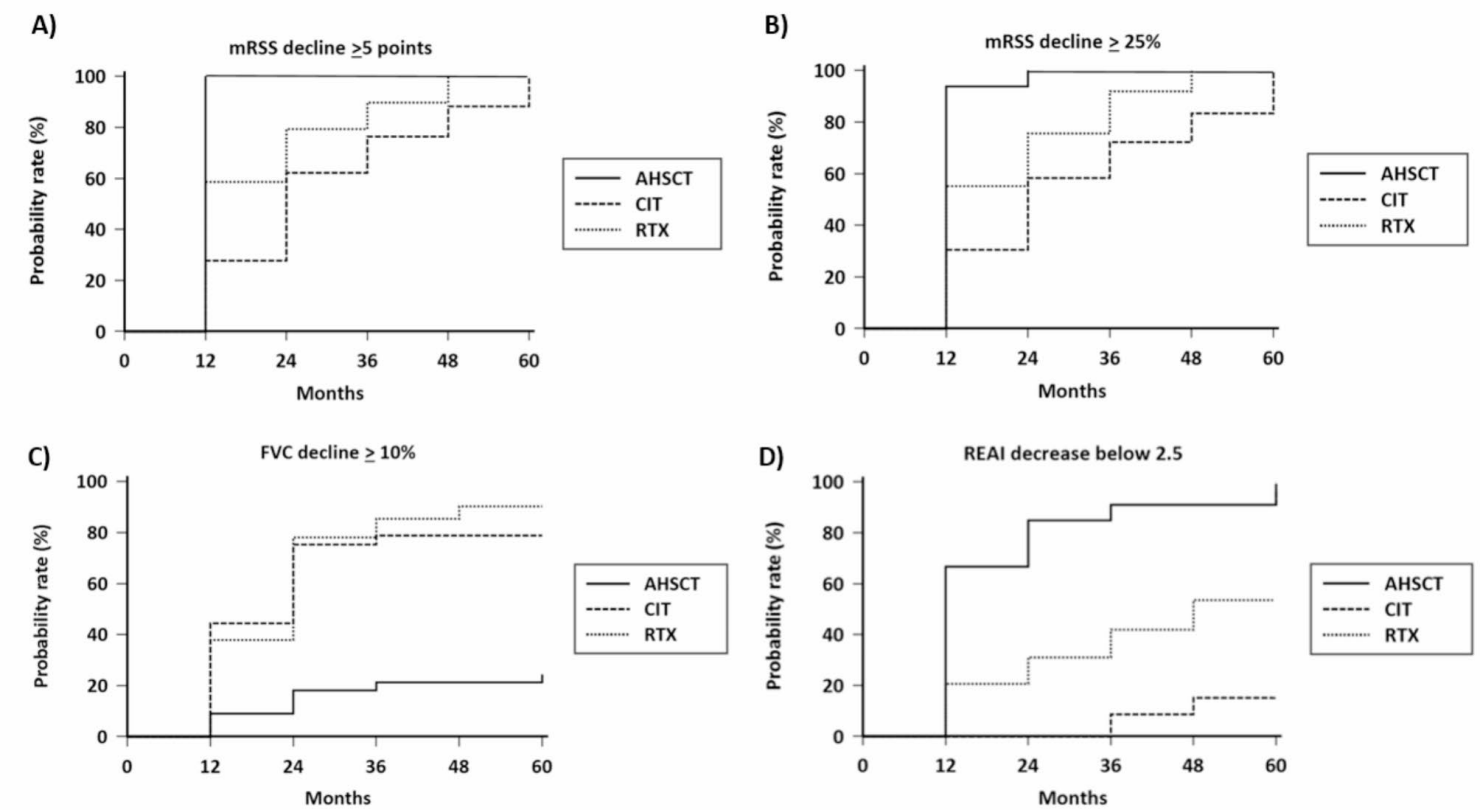
Percent probability of significant changes of the selected outcome measures (**a** and **b**: mRSS, **c**: FVC, **d**: REAI) in AHSCT-, RTX-, and CIT-treated patients. Detailed statistical results of log-rank test, HR and CI are reported in Table 2

Overall survival in the three groups of differently treated patients is shown in Fig. 1. The survival rate in the AHSCT-treated patients is significantly higher than what was observed in the other two groups. The overall survival is not statistically different between the RTX- and CIT-treated patients (see results of log-rank test in Table 2). The probability of a reduction of the mRSS of at least 5 points and of 25% or more are both significantly higher in transplanted patients in comparison with both patients treated with RTX and CIT. There is also a significant difference of this probability between the RTX- and CIT-treated patients, being the RTX therapeutical regimen slightly superior to CITs in improving skin involvement (Fig. 2a and b; Table 2 for detailed results).

The probability of a decline of lung function (decrease FVC ≥ 10% and combined decline of FVC ≥ 10% or DLCO ≥ 15% or both) was significantly higher in RTX- and CIT-treated groups in comparison with patients who underwent AHSCT. No significant difference in the lung function test decline was observed between RTX- and CIT-treated patients (Fig. 2c; Table 2).

The probability of reduction of REAI under 2.5 points is significantly higher in transplanted AHSCT patients in comparison with RTX- and CIT-treated groups. The probability of decline of this disease activity score was also significantly higher in patients treated with RTX in comparison with CIT-treated group (Fig. 2d; Table 2 for detailed results).

**Table 2:** Statistical analysis obtained comparing the Kaplan-Meier curves of the selected outcome measures in differently treated groups

Outcome measures	Therapeutic regimens	Log-rank test chi squared	Log-rank test significance (*p*)	HR value	HR value CI
Overall survival rate	RTX vs. AHSCT	7.25	< 0008	4.49	1.51–13.40
	CIT vs. AHSCT	19.72	< 0.0001	6.70	2.89–15.52
	CIT vs. RTX	3.30	0.069	-	-
Rate of reduction of mRSS of 5 points or more	AHSCT vs. RTX	16.21	< 0.0002	13.14	3.75–46.03
	AHSCT vs. CIT	36.87	< 0.0001	20,35	7.69–53.83
	RTX vs. CIT	5.12	< 0.03	2,45	1.13–5.33
Rate of reduction of mRSS of 25% or more	AHSCT vs. RTX	13.88	< 0.0003	7,72	2.63–22.61
	AHSCT vs. CIT	30.33	< 0.0001	13.08	5.24–32.65
	RTX vs. CIT	4.74	< 0.03	2.33	1.09–5.01
Rate of reduction of FVC at least of 10%*	RTX vs. AHSCT	32.41	< 0.0001	10.27	4.61–22.90
	CIT vs. AHSCT	22.30	< 0.0001	6.71	3.05–14.80
	CIT vs. RTX	0.48	0.49	-	-
Rate of reduction of REAI below 2.5 points	AHSCT vs. RTX	15.55	< 0.0002	4.50	2.13–9.51
	AHSCT vs. CIT	46.97	< 0.0001	17.89	7.84–40.83
	RTX vs. CIT	7.23	< 0.01	3.53	1.41–8.83

Table 2. Abbreviations: vs. versus, HR: Hazard Ratio, CI: Confidence Intervals. Note: The values of HR with CI are reported only in the cases in which the Log-rank test was significant

*The comparison of the reduction rate of combined FVC ≥ 10%/DLCO ≥ 15% in the three groups gives very similar results to that obtained considering FVC decline ≥ 10% alone [AHSCT vs. RTX: *p* < 0.0001, HR = 10.35 (CI 4.65–23.02); AHSCT vs. CIT: *p* < 0.0001, HR = 11.95 (CI 5.65–25.25); RTX vs. CIT not significant]. The results are the consequence of the fact that the numbers of patients having a decline of DLCO ≥ 15% but not a decline of FVC ≥ 10% were marginal in all three groups

### Changes in the mRSS and FVC with respect to baseline values during the follow-up

**Fig. 3:**
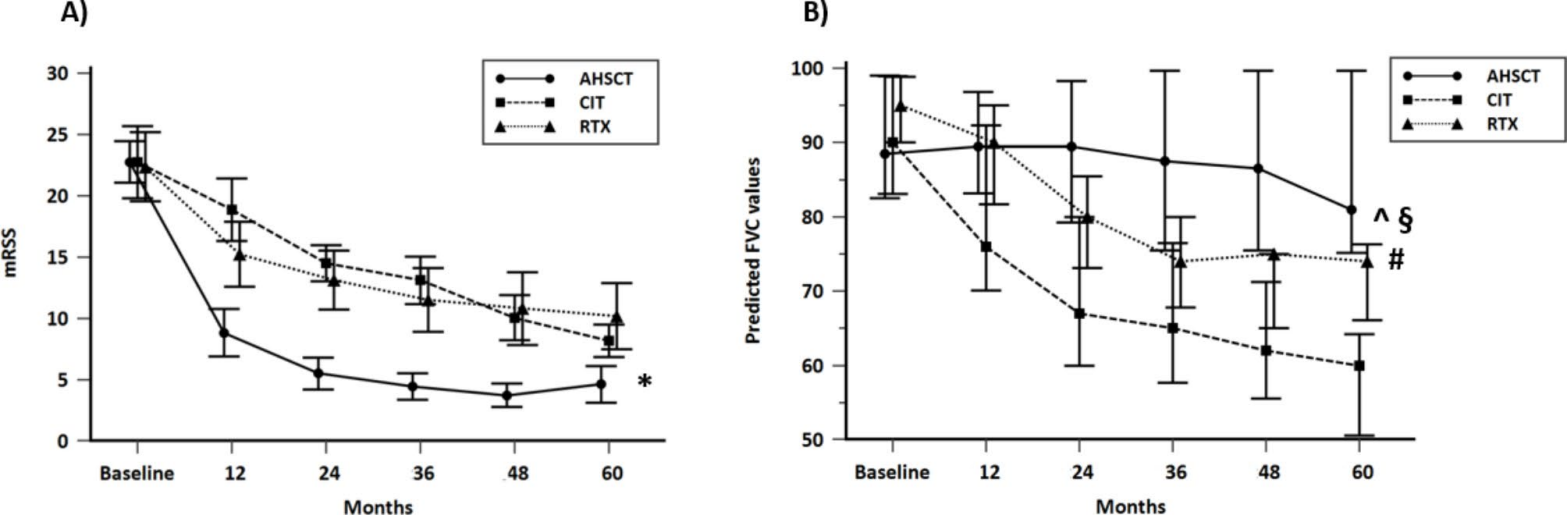
Overtime changes of mRSS (**a**) and FVC (**b**) during the 5 years follow-up, performed by the analysis of the variance with repeated measures (one-way ANOVA) in patients treated with AHSCT (continuous line), RTX (dotted line) and CIT (dot line). Patients who died during the follow-up were excluded by the analysis. Data are represented as mean values and 95% CI (vertical lines) in each observation time. (**a**). * *p* < 0.001 with respect to both RTX and CIT group. (**b**) ^ *p* < 0.05 with respect to RTX group; § *p* < 0.005 with respect to CIT group; # *p* < 0.05 with respect to CIT group

A generalized linear model with repeated measures obtained by one-way ANOVA was applied to evaluate the overtime changes of mRSS and FVC between the different treated groups. The results are graphically shown in Fig. 3a and b and the related statistical data reported in the legend. Briefly, overtime variation of mRSS values in AHSCT group are strongly significantly different from those observed in both patients treated with RTX and CIT. No significant difference of these overtime changes was observed between patients treated with RTX and CIT.

Overtime changes of FVC in both RTX and CIT groups were significantly different from those observed in AHSCT patients. A significant difference in FVC overtime changes was also present between patients treated with RTX in comparison with patients treated with CIT.

## Discussion

On the whole, the results of this study demonstrate that AHSCT procedure in patients with rapidly progressive dcSSc is more effective than RTX and CIT in improving the overall survival and in greatly decreasing the severity of skin thickness, measured by the mRSS, the level of disease activity, measured by a global assessment index, and in longer maintaining the lung function. RTX treatment is also more effective than CIT, although with a lower level of significance, in reducing skin impairment, disease activity, and in preserving lung function.

Finding an effective treatment for patients with dcSSc has been the object of numerous therapeutic studies in the past decades [6]. Different immunosuppressive agents have been tested in controlled trials for the treatment of this subset of patients with SSc, but contradictory results have been obtained. Namely, only one RCT was conducted with oral CYC, but this study failed to demonstrate any significant effect at two years in improving FVC in comparison with placebo, while it was found to induce a mild improvement of skin thickness and quality of life [23]. Two other RCTs were carried out comparing oral CYC with AZA plus low dose steroids, and with MMF. The first one, where the endpoints were represented by different lung function parameters, gave completely negative results in terms of differences between the two therapeutic regimens [24]. The second one indicated that both CYC and MMF were able to mildly improve FVC and to lower the mRSS in a comparable manner [25]. Only one large RCT was performed with MTX versus placebo in 2001, but this study failed to record any significant effectiveness in the treated group in terms of mRSS improvement [26].

The fact that AHSCT procedure in patients with a rapidly progressive form of dcSSc can be considered more effective than CIT is a widely confirmed statement. Three different trials (ASSIST, monocentric in USA, ASTIS, multicentric in Europe, SCOT multicentric in USA/Canada) comparing AHSCT with conventional intravenous CYC therapy have been published in the last decades [20, 27, 28]. The overall results can be summarized as follows: the mRSS greatly improved in the AHSCT treated patients in comparison with controls, who either improved to a lesser extent or worsened; lung function tests mildly improved in the transplanted patients and mildly worsened in the control group; quality of life improved in the AHSCT groups while it improved to a lesser extent or worsened in the CYC-treated patients. Mainly, at the fourth year of follow-up, the mortality rate was significantly lower in transplanted patients in the ASTIS and SCOT trials than in the CYC treated patients [20, 28].

Similar results were obtained in a retrospective case-control study performed by our group some years ago. In this study, we also reported a significant improvement of overall survival, mRSS and disease activity index in the AHSCT group, and a significantly more rapid decline of lung diffusion capacity in the CIT-treated control group [22].

In the present study, where the number of transplanted patients was expanded, the comparison with the historical group of CIT-treated patients reinforces the results already reported in our previous study and in published RCTs, again demonstrating the superiority of AHSCT procedure in rapidly decreasing the mRSS and the level of global disease activity, in longer maintaining lung function, and in drastically improving the mortality rate. At the end of the five-year follow-up, only 3 out of 33 patients died in the transplanted group in comparison to 22 out of 36 cases in the CIT-treated group. It is worth noting that the present study clearly shows that AHSCT procedure is also superior to RTX treatment when all the disease progression parameters were considered and also when the overall survival was recorded. In the RTX group death occurred in 11 out of 29 patients.

To our knowledge, this is the first study in which the comparison of AHSCT procedure and RTX treatment was head-to-head, including long-term results. A recently published study has shown the non-superiority of AHSCT adopted in 16 patients with respect to a combination therapy of RTX plus MMF, given to 21 patients. Apart from the fact that the two populations were quite small, and the follow-up was limited to 12 months, it is important to note that the RTX/MMF-treated group included 9 patients with lcSSc, a subset of patients that do not usually have a rapidly progressive disease [29].

Finally, our study also shows that RTX treatment works better than CIT in terms of reduction of global disease activity, skin impairment and longer preserving of lung function. This latter result was obtained only when FVC decline was evaluated by analysis of the variance with repeated measures, but not by the survival curve analysis.

It is difficult to make a comparison between the data of the present study and the previous trials in which RTX was compared with traditional immunosuppressive agents [30]. Most of these studies were carried out on a very limited number of patients [31–39], or included a consistent number of patients with lcSSc [36, 40–42], with FVC baseline values under 70% [33, 37–40, 42–46], and a follow-up time from 6 months and 2 years [31, 33–35, 37–39, 41, 44–46]. A long-term prospective study was conducted by Daoussis et al. in 33 patients (30 with dcSSc) who were treated with RTX and 18 patients receiving different types of CIT. Apart from the fact that the two populations differed in disease duration, a consistent number of patients received a combination therapy (RTX plus MMF), and a very limited number of patients completed the follow up of seven years. Nevertheless, the results of this trial suggest that RTX may be more effective than CIT in preserving lung function at both 2 and 7 years and in more rapidly reducing the mRSS [40].

In conclusion, in agreement with previous controlled studies, the present one confirms that AHSCT is more effective than CIT in treating patients with a rapidly progressive form of dcSSc in its early phase, quite speedily improving skin involvement, disease activity, decreasing mortality rate and maintaining lung function for a longer time. Similar significant differences are also observed when the AHSCT procedure is compared with RTX therapy. However, RTX therapy seems to offer some advantages with respect to traditional immunosuppressive agents in inducing a decline of the disease activity index, a rapid reduction of the mRSS, and in preserving the lung function.

Hot issues are still unsolved. Firstly, transplantation related mortality still exists, although significantly reduced. In this regard important progress has been made thanks to a better selection of patients at low risk for transplantation, which has been achieved by a more extensive preliminary evaluation of lung and cardiac performances [21], and to the improvements adopted in the different steps of transplantation procedure. Another question to be answered is how long the effects of AHSCT will last. Preliminary data indicate that the incidence of a disease relapse could happen between 4 and 6 years after transplantation [47]. To capture the moment of relapse we certainly need a better definition of it, and validated instruments to catch and measure this event. Finally, which therapeutic approach we should adopt for longer maintenance of the results of AHSCT and eventually to avoid or treat the relapse, is largely unknown. Further studies are certainly necessary to approach and solve these important unmet issues.

## Data Availability

The datasets used and/or analysed during the current study are available from the corresponding author on reasonable request.

## Abbreviations

ANA: Anti-nuclear antibodies
AHSCT: Autologous haematopoietic stem cell transplantation
AZA: Azathioprine
CIT: Traditional immunosuppressive therapies
CYC: Cyclophosphamide
dcSSc: Diffuse cutaneous systemic sclerosis
EF: Ejection fraction
FVC: Forced vital capacity
HRCT: High resolution computed tomography
IVIG: Intravenous immunoglobulins
lcSSc: Limited cutaneous systemic sclerosis
MMF: Mycophenolate mofetil
mRSS: Modified Rodnan skin score
MTX: Methotrexate
PAPs: Systolic pulmonary artery pressure
REAI: Revised EUSTAR Activity Index
RTX: Rituximab
SSc: Systemic sclerosis

## References

[1] Denton CP, Khanna D. Systemic sclerosis. Lancet. 2017;390:1685–99. 28413064 10.1016/S0140-6736(17)30933-9

[2] Steen VD, Medsger TA. Changes in causes of death in systemic sclerosis, 1972–2002. Ann Rheum Dis. 2007;66:940–4. 17329309 10.1136/ard.2006.066068 PMC1955114

[3] Rubio-Rivas M, Royo C, Simeón CP, Corbella X, Fonollosa V. Mortality and survival in systemic sclerosis: systematic review and meta-analysis. Semin Arthritis Rheum. 2014;44:208–19. 24931517 10.1016/j.semarthrit.2014.05.010

[4] Tyndall AJ, Bannert B, Vonk M, Airò P, Cozzi F, Carreira PE, et al. Causes and risk factors for death in systemic sclerosis: a study from the EULAR Scleroderma trials and Research (EUSTAR) database. Ann Rheum Dis. 2010;69:1809–15. 20551155 10.1136/ard.2009.114264

[5] Domsic RT, Rodriguez-Reyna T, Lucas M, Fertig N, Medsger TA. Skin thickness progression rate: a predictor of mortality and early internal organ involvement in diffuse scleroderma. Ann Rheum Dis. 2011;70:104–9. 20679474 10.1136/ard.2009.127621 PMC3881170

[6] Pope JE, Denton CP, Johnson SR, Fernandez-Codina A, Hudson M, Nevskaya T. State-of-the-art evidence in the treatment of systemic sclerosis. Nat Rev Rheumatol. 2023;19:212–26. 36849541 10.1038/s41584-023-00909-5 PMC9970138

[7] Khanna D, Denton CP, Jahreis A, van Laar JM, Frech TM, Anderson ME, et al. Safety and efficacy of subcutaneous tocilizumab in adults with systemic sclerosis (faSScinate): a phase 2, randomised, controlled trial. Lancet. 2016;387:2630–40. 27156934 10.1016/S0140-6736(16)00232-4

[8] Khanna D, Lin CJF, Furst DE, Goldin J, Kim G, Kuwana M, et al. Tocilizumab in systemic sclerosis: a randomised, double-blind, placebo-controlled, phase 3 trial. Lancet Respir Med. 2020;8:963–74. 32866440 10.1016/S2213-2600(20)30318-0

[9] Maher TM, Tudor VA, Saunders P, Gibbons MA, Fletcher SV, Denton CP, et al. Rituximab versus intravenous cyclophosphamide in patients with connective tissue disease-associated interstitial lung disease in the UK (RECITAL): a double-blind, double-dummy, randomised, controlled, phase 2b trial. Lancet Respir Med. 2023;11:45–54. 36375479 10.1016/S2213-2600(22)00359-9

[10] Distler O, Highland KB, Gahlemann M, Azuma A, Fischer A, Mayes MD, et al. Nintedanib for systemic sclerosis–Associated interstitial lung disease. N Engl J Med. 2019;380:2518–28. 31112379 10.1056/NEJMoa1903076

[11] Snowden JA, Sánchez-Ortega I, Corbacioglu S, Basak GW, Chabannon C, de la Camara R, et al. Indications for haematopoietic cell transplantation for haematological diseases, solid tumours and immune disorders: current practice in Europe, 2022. Bone Marrow Transpl. 2022;57:1217–39. 10.1038/s41409-022-01691-w PMC9119216 35589997

[12] Kowal-Bielecka O, Fransen J, Avouac J, Becker M, Kulak A, Allanore Y, et al. Update of EULAR recommendations for the treatment of systemic sclerosis. Ann Rheum Dis. 2017;76:1327–39. 27941129 10.1136/annrheumdis-2016-209909

[13] Xue E, Minniti A, Alexander T, Del Papa N, Greco R. Cellular-based therapies in systemic sclerosis: from hematopoietic stem cell transplant to innovative approaches. Cells. 2022;11:3346. 36359742 10.3390/cells11213346 PMC9658618

[14] Bruera S, Sidanmat H, Molony DA, Mayes MD, Suarez-Almazor ME, Krause K et al. Stem cell transplantation for systemic sclerosis. Cochrane Database of Systematic Reviews 2022, Issue 7., No.:CD011819 10.1002/14651858.CD011819.pub2 PMC9336163 35904231

[15] Preliminary criteria for the classification of systemic sclerosis (scleroderma). Subcommittee for scleroderma criteria of the American Rheumatism Association Diagnostic and Therapeutic Criteria Committee. Arthritis Rheum. 1980;23:581–90. 7378088 10.1002/art.1780230510

[16] van den Hoogen F, Khanna D, Fransen J, Johnson SR, Baron M, Tyndall A, et al. 2013 classification criteria for systemic sclerosis: an American college of rheumatology/European league against rheumatism collaborative initiative. Ann Rheum Dis. 2013;72:1747–55. 24092682 10.1136/annrheumdis-2013-204424

[17] Dobrota R, Maurer B, Graf N, Jordan S, Mihai C, Kowal-Bielecka O, Allanore Y, Distler O. EUSTAR coauthors. Prediction of improvement in skin fibrosis in diffuse cutaneous systemic sclerosis: a EUSTAR analysis. Ann Rheum Dis 2016:1743–8. 10.1136/annrheumdis-2015-208024 PMC5036205 27016052

[18] Petelytska L, Bonomi F, Cannistrà C, Fiorentini E, Peretti S, Torracchi S, Bernardini P, Coccia C, De Luca R, Economou A, Levani J, Matucci-Cerinic M, Distler O, Bruni C. Heterogeneity of determining disease severity, clinical course and outcomes in systemic sclerosis-associated interstitial lung disease: a systematic literature review. RMD Open. 2023;9(4):e003426. 37940340 10.1136/rmdopen-2023-003426 PMC10632935

[19] Valentini G, Iudici M, Walker UA, Jaeger VK, Baron M, Carreira P, et al. The European Scleroderma Trials and Research group (EUSTAR) task force for the development of revised activity criteria for systemic sclerosis: derivation and validation of a preliminarily revised EUSTAR activity index. Ann Rheum Dis. 2017;76:270–6. 27621285 10.1136/annrheumdis-2016-209768

[20] Van Laar JM, Farge D, Sont JK, Naraghi K, Marjanovic Z, Larghero J, et al. Autologous hematopoietic stem cell transplantation vs intravenous pulse cyclophosphamide in diffuse cutaneous systemic sclerosis: a Randomized Clinical Trial. JAMA. 2014;311:2490. 25058083 10.1001/jama.2014.6368

[21] Farge D, Burt RK, Oliveira M-C, Mousseaux E, Rovira M, Marjanovic Z, et al. Cardiopulmonary assessment of patients with systemic sclerosis for hematopoietic stem cell transplantation: recommendations from the European Society for Blood and Marrow Transplantation Autoimmune Diseases Working Party and collaborating partners. Bone Marrow Transpl. 2017;52:1495–503. 10.1038/bmt.2017.56 PMC5671927 28530671

[22] Del Papa N, Onida F, Zaccara E, Saporiti G, Maglione W, Tagliaferri E, et al. Autologous hematopoietic stem cell transplantation has better outcomes than conventional therapies in patients with rapidly progressive systemic sclerosis. Bone Marrow Transpl. 2017;52:53–8. 10.1038/bmt.2016.211 27548467

[23] Tashkin DP, Elashoff R, Clements PJ, Goldin J, Roth MD, Furst DE, et al. Cyclophosphamide versus Placebo in Scleroderma Lung Disease. N Engl J Med. 2006;354:2655–66. 16790698 10.1056/NEJMoa055120

[24] Hoyles RK, Ellis RW, Wellsbury J, Lees B, Newlands P, Goh NSL, et al. A multicenter, prospective, randomized, double-blind, placebo-controlled trial of corticosteroids and intravenous cyclophosphamide followed by oral azathioprine for the treatment of pulmonary fibrosis in scleroderma. Arthritis Rheum. 2006;54:3962–70. 17133610 10.1002/art.22204

[25] Tashkin DP, Roth MD, Clements PJ, Furst DE, Khanna D, Kleerup EC, et al. Mycophenolate mofetil versus oral cyclophosphamide in scleroderma-related interstitial lung disease (SLS II): a randomised controlled, double-blind, parallel group trial. Lancet Respiratory Med. 2016;4:708–19. 10.1016/S2213-2600(16)30152-7 PMC5014629 27469583

[26] Pope JE, Bellamy N, Seibold JR, Baron M, Ellman M, Carette S, et al. A randomized, controlled trial of methotrexate versus placebo in early diffuse scleroderma. Arthritis Rheum. 2001;44:1351–8. 11407694 10.1002/1529-0131(200106)44:6<1351::AID-ART227>3.0.CO;2-I

[27] Burt RK, Shah SJ, Dill K, Grant T, Gheorghiade M, Schroeder J, et al. Autologous non-myeloablative haemopoietic stem-cell transplantation compared with pulse cyclophosphamide once per month for systemic sclerosis (ASSIST): an open-label, randomised phase 2 trial. Lancet. 2011;378:498–506. 21777972 10.1016/S0140-6736(11)60982-3

[28] Sullivan KM, Goldmuntz EA, Keyes-Elstein L, McSweeney PA, Pinckney A, Welch B, et al. Myeloablative autologous stem-cell transplantation for severe Scleroderma. N Engl J Med. 2018;378:35–47. 29298160 10.1056/nejmoa1703327 PMC5846574

[29] Keret S, Henig I, Zuckerman T, Kaly L, Shouval A, Awisat A et al. Outcomes in progressive systemic sclerosis treated with autologous hematopoietic stem cell transplantation compared with combination therapy. Rheumatology. 2023;1534–38 10.1093/rheumatology/kead457 37672021

[30] Moradzadeh M, Aghaei M, Mehrbakhsh Z, Arab-Bafrani Z, Abdollahi N. Efficacy and safety of rituximab therapy in patients with systemic sclerosis disease (SSc): systematic review and meta-analysis. Clin Rheumatol. 2021;40:3897–918. 33796953 10.1007/s10067-021-05698-4

[31] Lafyatis R, Kissin E, York M, Farina G, Viger K, Fritzler MJ, et al. B cell depletion with rituximab in patients with diffuse cutaneous systemic sclerosis. Arthritis Rheum. 2009;60:578–83. 19180481 10.1002/art.24249 PMC2637937

[32] Bosello S, De Santis M, Lama G, Spanò C, Angelucci C, Tolusso B, et al. B cell depletion in diffuse progressive systemic sclerosis: safety, skin score modification and IL-6 modulation in an up to thirty-six months follow-up open-label trial. Arthritis Res Ther. 2010;12:R54. 20338043 10.1186/ar2965 PMC2888203

[33] Daoussis D, Liossis S-NC, Tsamandas AC, Kalogeropoulou C, Kazantzi A, Sirinian C, et al. Experience with rituximab in scleroderma: results from a 1-year, proof-of-principle study. Rheumatology (Oxford). 2010;49:271–80. 19447770 10.1093/rheumatology/kep093 PMC2806066

[34] Smith V, Piette Y, van Praet JT, Decuman S, Deschepper E, Elewaut D, De Keyser F. Two-year results of an open pilot study of a 2-treatment course with rituximab in patients with early systemic sclerosis with diffuse skin involvement. J Rheumatol. 2013;40(1):52–7. 23118116 10.3899/jrheum.120778

[35] Melsens K, Vandecasteele E, Deschepper E, Badot V, Blockmans D, Brusselle G, De Langhe E, De Pauw M, Debusschere C, Decuman S, Deroo L, Houssiau F, Lenaerts J, Piette Y, Thevissen K, Vanthuyne M, Westhovens R, Wijnant S, De Keyser F, Smith V. Two years follow-up of an open-label pilot study of treatment with rituximab in patients with early diffuse cutaneous systemic sclerosis. Acta Clin Belg. 2018;73(2):119–25. 28891418 10.1080/17843286.2017.1372244

[36] Giuggioli D, Lumetti F, Colaci M, Fallahi P, Antonelli A, Ferri C. Rituximab in the treatment of patients with systemic sclerosis. Our experience and review of the literature. Autoimmun Rev. 2015;14:1072–8. 26209905 10.1016/j.autrev.2015.07.008

[37] Ebata S, Yoshizaki A, Fukasawa T, Miura S, Takahashi T, Sumida H, et al. Rituximab therapy is more effective than cyclophosphamide therapy for Japanese patients with anti-topoisomerase I-positive systemic sclerosis-associated interstitial lung disease. J Dermatol. 2019;46:1006–13. 31502326 10.1111/1346-8138.15079

[38] Vilela VS, Maretti GB, Gama LM da, Costa S, Rufino CH, Levy RL. RA. Rituximab for the therapy of systemic sclerosis: a series of 10 cases in a single center. Revista Brasileira de Reumatologia (English Edition). 2016;56:458–63. 10.1016/j.rbre.2016.06.003 27692396

[39] Thiebaut M, Launay D, Rivière S, Mahévas T, Bellakhal S, Hachulla E, et al. Efficacy and safety of rituximab in systemic sclerosis: French retrospective study and literature review. Autoimmun Rev. 2018;17:582–7. 29635080 10.1016/j.autrev.2017.12.010

[40] Daoussis D, Melissaropoulos K, Sakellaropoulos G, Antonopoulos I, Markatseli TE, Simopoulou T, et al. A multicenter, open-label, comparative study of B-cell depletion therapy with Rituximab for systemic sclerosis-associated interstitial lung disease. Semin Arthritis Rheum. 2017;46:625–31. 27839742 10.1016/j.semarthrit.2016.10.003

[41] Elhai M, Boubaya M, Distler O, Smith V, Matucci-Cerinic M, Sancho JJA, et al. Outcomes of patients with systemic sclerosis treated with rituximab in contemporary practice: a prospective cohort study. Ann Rheum Dis. 2019;78:979–87. 30967395 10.1136/annrheumdis-2018-214816

[42] Jordan S, Distler JHW, Maurer B, Huscher D, Van Laar JM, Allanore Y, et al. Effects and safety of rituximab in systemic sclerosis: an analysis from the European Scleroderma Trial and Research (EUSTAR) group. Ann Rheum Dis. 2015;74:1188–94. 24442885 10.1136/annrheumdis-2013-204522

[43] Bosello SL, De Luca G, Rucco M, Berardi G, Falcione M, Danza FM, et al. Long-term efficacy of B cell depletion therapy on lung and skin involvement in diffuse systemic sclerosis. Semin Arthritis Rheum. 2015;44:428–36. 25300701 10.1016/j.semarthrit.2014.09.002

[44] Daoussis D, Liossis S-NC, Tsamandas AC, Kalogeropoulou C, Paliogianni F, Sirinian C, et al. Effect of long-term treatment with rituximab on pulmonary function and skin fibrosis in patients with diffuse systemic sclerosis. Clin Exp Rheumatol. 2012;30:S17–22. 22244622

[45] Sircar G, Goswami RP, Sircar D, Ghosh A, Ghosh P. Intravenous cyclophosphamide vs rituximab for the treatment of early diffuse scleroderma lung disease: open label, randomized, controlled trial. Rheumatology (Oxford). 2018;57:2106–13. 30053212 10.1093/rheumatology/key213

[46] Lepri G, Avouac J, Airò P, Anguita Santos F, Bellando-Randone S, Blagojevic J, et al. Effects of Rituximab in connective tissue disorders related interstitial lung disease. Clin Exp Rheumatol. 2016;34(Suppl 100):181–5. 27749242

[47] Henes J, Oliveira MC, Labopin M, Badoglio M, Scherer HU, Del Papa N, et al. Autologous stem cell transplantation for progressive systemic sclerosis: a prospective non-interventional study from the European Society for Blood and Marrow Transplantation Autoimmune Disease Working Party. Haematologica. 2020;106:375–83. 10.3324/haematol.2019.230128 PMC7849556 31949011

